# Nimotuzumab combined with concurrent chemoradiotherapy in locally advanced nasopharyngeal carcinoma: a retrospective analysis

**DOI:** 10.18632/oncotarget.8225

**Published:** 2016-03-21

**Authors:** Zhi-gang Liu, Yu Zhao, Jiao Tang, Yu-juan Zhou, Wen-juan Yang, Yan-fang Qiu, Hui Wang

**Affiliations:** ^1^ Key Laboratory of Translational Radiation Oncology, Department of Radiation Oncology, Hunan Cancer Hospital, The Affiliated Cancer Hospital of Xiangya School of Medicine, Central South University, Changsha, Hunan, China

**Keywords:** NPC, nimotuzumab, chemoradiotherapy, EGFR

## Abstract

Nimotuzumab is a blocking monoclonal antibody against epidermal growth factor receptor (EGFR). However, little is known about the safety and preliminary efficacy of nimotuzumab combined with concurrent chemoradiotherapy in locally advanced NPC patients. A total of 42 patients diagnosed between 2011 and 2013 were enrolled. Our results demonstrated 38 patients had a complete response (90.5%), 4 patients had a partial response (9.5%). And no patients had progressive disease at early treatment response evaluation, giving an ORR of 100%. The 2-year local recurrence-free survival (LRFS), distant metastasis-free survival (DMFS) and overall survival (OS) were 96.4%, 93.1% and 96.6% respectively. The most common adverse events were mucositis (19 patients), hematology toxicity (14 patients) with 6 and 3 cases of grade 3/4 toxicity respectively. Skin rash was not developed in our 43 patients. Thus, nimotuzumab combined with concurrent chemoradiotherapy showed encouraging outcomes in the treatment of locally advanced nasopharyngeal carcinoma, without accumulation of toxicity and well-tolerated.

## INTRODUCTION

Nasopharyngeal carcinoma (NPC) is a highly invasive malignancy that rises from the epithelial lining of the nasopharynx. With an estimated 86,700 new cases and 50,800 deaths in 2012, NPC is viewed as a relatively rarer form of cancer globally. But in endemic regions, including Southern China and Southeast Asia, the incidence rate reached 15-50/100000 [1]. Radiotherapy (RT) is the main treatment for NPC and its clinical outcome has been greatly improved since the application of intensity-modulated radiotherapy (IMRT) [2]. Although this tumor is sensitive to radiotherapy, therapy may fail in patients with advanced stage disease, as the disease is highly invasive and metastatic in nature [2, 3]. The treatment outcome for locally advanced NPC patients remains to be further improved.

At the forefront of research are therapies involving molecular targets such as epidermal growth factor receptor (EGFR), which is a topic researched extensively over the last decade. EGFR overexpression has been observed in many different cancers, including gliomas, sarcomas, and head and neck cancers [4]. Several inhibitors of the EGFR, e.g., cetuximab, panitumumab, erlotinib, and gefitinib, have shown favorable results in clinical trials [5–8]. For NPC, cetuximab with concurrent chemoradiotherapy is tolerable and has shown promising advantage for NPC prognosis [9].

Nimotuzumab is a blocking monoclonal antibody against EGFR without intrinsic stimulating activity [10]. Compared with other anti-EGFR antibodies, nimotuzumab displayed a longer half-life and elevated area under the curve than cetuximab at the same dose level [11]. Besides, safety data showed that nimotuzumab rarely caused severe dermatological toxicity, which is the most common adverse events resulting from cetuximab and panitumumab. Based on this, it is expected to improve the quality of life [12]. Nimotuzumab currently has marketing approval for the treatment of advanced head and neck, glioma, and esophageal cancer patients in combination with irradiation or chemoradiotherapy [13].

EGFR is overexpressed in 80% of NPC patients and its expression is associated with unfavorable T stage and overall survival [14]. However, little is known about the application of nimotuzumab in NPC patients. Here, we retrospectively studied the safety and preliminary efficacy of nimotuzumab combined with radiotherapy and chemotherapy in locally advanced NPC patients.

## RESULTS

### Patient characteristics

Between November 2011 and November 2013, 42 consecutive patients with locally advanced NPC were treated with nimotuzumab plus concurrent chemoradiotherapy. Until September 30, 2015, the median follow-up time was 25 months (range: 7–44 months). Characteristics of patients are shown in Table 1. The median patient age was 46 years (range: 21–71 years). All patients received cisplatin based concurrent chemotherapy. Among these patients, 27 received concurrent chemotherapy, 7 received induction plus concurrent chemotherapy, 8 received induction, concurrent plus adjuvant chemotherapy. The general course was four to six cycles. All patients received nimotuzumab and the range of cycles of nimotuzumab treatment was four to seven cycles.13 patients received 100mg/week nimotuzumab and 29 patients received 200mg/week nimotuzumab.

**Table 1:** Baseline demographics and clinical characteristics

Characteristics	No. of patients
Total	42
Sex	
Men	28
Women	14
Age (years)Median (range)	46(21–71)
≤65	40
>65	3
Histopathology (WHO type)	
Type 1	4
Types 2.1 & 2.2	38
T-classification	
T1	4
T2	11
T3	11
T4	16
N-classification	
N0	2
N1	6
N2	23
N3	11
Stage	
III	18
IV	24
Chemotherapy	
Concurrent	27
Induction + concurrent	7
Induction + concurrent + adjuvant	8
Chemotherapy regimen	
TP	29
PF	11
GP	2

### Efficacy

**Figure 1:**
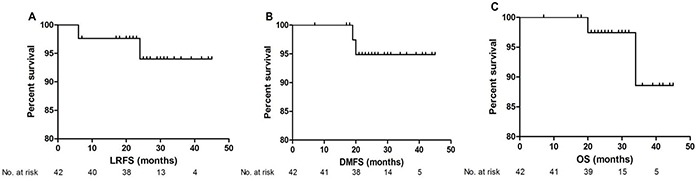
Kaplan–Meier estimates of local recurrence free survival **A.** distant metastasis free survival **B.** and overall survival **C.**

Of the 42 patients, 9.5% (4/42) and 4.8% (2/42) of patients had experienced progression or died at the last follow-up. The 2 patients died because of distant metastasis, other than treatment related toxicities. The response rates were as follows: 38 patient had a complete response (90.5%), 4 patients had a partial response (9.5%), and no patients had progressive disease, giving an objective response rate (ORR) of 100% (Table 2). The 2-year LRFS, DMFS and OS were 96.4%, 93.1% and 96.6% respectively (Figure 1).

**Table 2:** Response to treatment

Response	Number(%)
Complete response (CR)	38 (90.5)
Partial response (PR)	4(9.5)
Stable disease (SD)	0 (0)
Progression disease (PD)	0 (0)
Objective response (CR+PR)	42 (100.0)

### Safety and toxicity

The 42 patients received 254 cycles of nimotuzumab. Detailed adverse effects are displayed in Table 3. In general, patients were well tolerated and there were no treatment-related deaths. The most common adverse events were mucositis (19 patients) and hematology toxicity (14 patients), with 6 and 3 cases of grade 3/4 toxicity respectively. The adverse event most likely associated with nimotuzumab is skin rash which did not develop in our 42 patients. No long-term drug-related toxicity was seen during the median follow-up of 25 months.

**Table 3:** Toxicity of nimotuzumab plus chemoradiotherapy

Adverse events	All grades, N	Grade 3/4, N (%)
Radiodermatitis	4	1(20)
Mucositis	19	6(24)
Nausea/vomiting	8	0(0)
Leukocytopenia	18	3(16.7%)
Anemia	11	0(0)
Thrombocytopenia	14	0(0)

## DISCUSSION

The epidermal growth factor receptor (EGFR) plays an important role in tumorigenesis and maintenance of cancers, making it a possible therapeutic target for cancer treatment. Epidermal growth factor receptors (EGFR) are overexpressed in a wide range of malignancies including head and neck, colon, and breast cancers [15]. EGFR is overexpressed in 80% of NPC patients [14], which shed important new light on the utilization of the monoclonal antibody against EGFR.

Nimotuzumab (h-R3) is a humanized IgG1 isotype monoclonal antibody. It is approved for the following indications: for nasopharyngeal cancer in China; for squamous cell carcinoma in head and neck (SCCHN) in India, Cuba, Argentina, Colombia, Ivory Coast, Gabon, Ukraine, Peru and Sri Lanka; for pediatric and adult glioma in Cuba, Argentina, Philippines and Ukraine; orphan drug status for glioma in USA and for glioma and pancreatic cancer in Europe [16]. Owing to the limited indications for nasopharyngeal carcinoma, there were little publications to assess the efficiency and safety of nimotuzumab combined with concurrent chemoradiotherapy in locally advanced nasopharyngeal carcinoma worldwide except China. For example, some studies have investigated the efficiency and safety of nimotuzumab in other kinds of tumors. Data from clinical phase II studies have demonstrated that nimotuzumab could be safely combined with radiotherapy plus concurrent capecitabine (pCR=19.0 %) in locally advanced rectal cancer [17]. It also suggested that EGFR inhibitor in combination with chemoradiotherapy in patients with hypopharyngeal carcinoma was well tolerated and resulted in encouraging laryngeal preservation survival rate. The 3-year local control survival, disease-free survival, overall survival and laryngeal preservation survival rates were 66.8%, 59.0%, 68.9% and 86.7%, respectively. The most common grade 3 or higher side effect was oropharyngeal mucositis [18]. There was also a trend towards improved treatment efficacy of chemoradiotherapy combined with nimotuzumab against malignant gliomas. The one year survival rates of the treatment and control groups were 81.3% and 69.1%, respectively (P>0.05) [19]. Likewise, the similar objective response of nimotuzumab plus chemoradiotherapy was demonstrated in epithelial origin esophageal tumors [20].

In terms of monoclonal antibody against EGFR and nasopharyngeal carcinoma, an early study showed that radiotherapy plus cetuximab improved locoregional control, overall survival (49.0 months vs 29.3 months) and reduced mortality (hazard ratio for death, 0.74; P=0.03) without increasing the common toxic effects of the head and neck cancers [21]. In locally advanced nasopharyngeal carcinoma, it was previously reported that, using IMRT combined with concurrent chemotherapy, 1- and 3-year local control (LC) rates, distant control (DC) rates, overall survival (OS) rates were 98% and 90%, 92% and 86%, 96% and 82% [22], respectively. Our data were higher than the above rates, which implied nimotuzumab could harvest a probable curative effect. However, it requires further evaluation by longer follow up period and a larger sample size. In addition, our results demonstrated no nimotuzumab related skin rash was reported in our 42 patients, which could be explained by the property that nimotuzumab has lesser affinity and hence binds with less avidity than other monoclonal EGFR antibodies (cetuximab and panitumumad). Thus, it spares healthy tissues and avoids the severe toxicities. It has been experimentally observed that anti-EGFR tyrosine kinase inhibitors are known to inhibit radioresistance by various mechanisms. Firstly, there was a dose dependent inhibition of vascular endothelial growth factor (VEGF) expression inhibition by nimotuzumab [16]. It could increase apoptosis by inhibiting different signal transduction hubs, such as mitogen-activated protein kinase (MAPK), signal transducer and activator of transcription 3(pSTAT3), phosphatidylinositol 3-kinase (PI3K), protein kinase B (Akt/PKB) [23]. Also, it could induce autophagy activation [24]. Nimotuzumab could upregulate insulin-like growth factor binding protein-3 (IGFBP-3), which could increase chemoradiosensitivity [25, 26]. G_1_ and G_2_ phases are relatively high sensitive to radiotherapy. Nimotuzumab could induce the G_0_/G_1_ arrest and G_2_/M phase arrest [17]. Nimotuzumab could also be cytolytic on target tumors by its capacity to cause antibody dependent cell mediated cytotoxicity (ADCC) and complement dependent cytotoxicity (CDC) [27].

However, due to the small number of patients in this trial, further random control III phase clinical research is required to confirm these conclusions. And we expect the promising result as quickly as possible from a clinical trial of nimotuzumab in combination with chemoradiation for locally advanced nasopharyngeal cancer, even though this study is ongoing but not recruiting participants (ClinicalTrials.gov: NCT01074021). We also look forward to the result from another clinical trial that Phase III Study of neoadjuvant TPF chemotherapy followed by radiotherapy and concurrent nimotuzumab or cisplatin for locoregionally advanced nasopharyngeal carcinoma (NPC) (ClinicalTrials.gov: NCT02012062) with the purpose to evaluate whether concurrent nimotuzumab could decrease the severe acute treatment-related toxicities. Sencondly, since nimotuzumab is more expensive than conventional treatment, it is urgent to classify the subgroup who could gain most from this regiment by finding meaningful clinical prediction molecule or detecting EGFR, KRAS gene level before treatment. Thirdly, four patients received only 4 weeks of nimotuzumab due to economic issues. China is a developing country with obvious economic imbalance. Many people cannot afford this expensive drug. In addition, we will further report longer follow-up data to warrant the conclusion.

In summary, we present the first evidence that nimotuzumab combined with concurrent chemoradiotherapy in the treatment of local advanced nasopharyngeal carcinoma can achieve a better short-term effect without accumulation of toxicity and is well-tolerated.

## MATERIALS AND METHODS

### Patients selection

Between November 2011 and November 2013, 42 consecutive patients with NPC in the Department of Radiotherapy, the Affiliated Cancer Hospital of Xiangya School of Medicine (Central South University, Changsha, China), were enrolled in this study, which had the following criteria:(1) histologically confirmed nasopharyngeal squamous-cell carcinoma;(2) age ranges from 21 to 71 (3) Karnofsky performance status (KPS) ≥60;(4) a life expectancy of greater than six months;(5) leukocyte count ≥4.0×10^9^/L, absolute neutrophil count ≥1.5×10^9^/L, platelets ≥100×10^9^ /L, serum creatinine <1.4 mg/dl and total bilirubin ≤1.2 mg/dL; (6) provided written informed consent. Patients with history of prior malignancy, prior chemotherapy, immunotherapy, radiotherapy, evidence of distant metastasis or a concurrent secondary malignancy, pregnancy or lactation were excluded from the study.

### Radiotherapy

All patients received intensity modulation radiated therapy. Subjects were immobilized in a supine position with the head in a neutral position with a tailored thermoplastic mask covering the head, neck, and shoulders. Intravenous contrast-enhanced CT using 2.5 mm slice from the vertex to the manubriosternal joint was performed for planning. Megavoltage photons (6 MV) were used to treat the primary tumor and involved lymph nodes. The plans were designed and optimized using the Pinnacle inverse planning system. The prescribed radiation dose was 70-74 Gy at 2.25 Gy per fraction delivered to the PGTVnx, and 60-64 Gy at 2.00 Gy per fraction delivered to the PTV1. The PTV2 was treated to 54-56Gy at 1.8 Gy per fractions. All patients were treated once daily, five fractions weekly. Dose constrains to the critical structures were within the tolerance according to the RTOG 0225 protocol, and efforts were made to meet the criteria as closely as possible.

### Antibody therapy and chemotherapy

Nimotuzumab was administered at 100 or 200mg weekly throughout RT weekly. All patients received cisplatin based induction and/or concurrent chemotherapy. Cisplatin was delivered at 75 mg/m^2^ every three weeks. The chemotherapy regimen included TP (docetaxel 60 mg/m2/day on day 1, and cisplatin 25 mg/m2/day on days 1–3), PF (cisplatin 25 mg/m2/day on days 1–3, and 5-fluorouracil 500 mg/m2/day on days 1–3) and GP regimen (gemcitabine 1,000 mg/m2/day on days 1 and 8, cisplatin25 mg/m2/day on days 1–3). Patients were monitored weekly by performing clinical examination, blood count and biochemistry analyses, including liver function. The National Cancer Institute Common Toxicity Criteria version 3.0 was used to assess grade toxicities. If a toxicity (hematological or gastrointestinal) was considered to be primarily related to chemotherapy and occurred at >Grade 2, chemotherapy was discontinued until the toxicity resolved to Grade 0–1, then chemotherapy was reinitiated.

### Response and toxicity assessment

Short-term efficacy was assessed 3 months after the completion of radiotherapy. The residual tumor and lymph nodes were evaluated according to clinical examinations (including nasopharyngeal endoscopy) and MRI or Positron emission tomography computed tomography (PET-CT) if necessary. We used Response Evaluation Criteria in Solid Tumors version 1.1 (RECIST v1.1) to assess the early treatment response. Acute toxicities were evaluated according to the Common Toxicity Criteria 3.0 of the American National Cancer Institute. Toxicities assessment were performed once a week during radiotherapy and late toxicity (occurring > 90 days) was conducted at least once every 3 months after the end of radiotherapy.

### Follow up

Patients were followed up at 3 months post-completion, and then every 3 months for the first 3 years and every 6 months thereafter. The primary end point was overall survival and adverse reaction. Overall survival is defined as the time (in months) from the date of admission to the date of death from any causes or the last follow-up. The second end point was local-recurrence-free survival (LRFS) and distant metastasis free survival (DMFS). LRFS is defined as the time (in months) from the date of admission until the date of local or regional failure with pathology confirmed. DMFS is defined as the time (in months) from the date of admission to the date of distant metastasis.

### Statistical analysis

Analyses were performed using SPSS 18.0 software. Quantitative data were expressed as median. Survival curves were analyzed by the method of Kaplan–Meier. Log-rank tests were used to detect differences between the groups. P value < 0.05 was considered significant.
